# Evaluation of Microbubbles as Contrast Agents for Ultrasonography and Magnetic Resonance Imaging

**DOI:** 10.1371/journal.pone.0034644

**Published:** 2012-04-10

**Authors:** Ling Li, Qiang Wei, Hong-Bo Li, Song Wen, Gao-Jun Teng

**Affiliations:** 1 Jiangsu Key Laboratory of Molecular Imaging and Functional Imaging, Department of Radiology, Zhong-Da Hospital, Medical School of Southeast University, Nanjing, China; 2 Department of Ultrasound, the Second Affiliated Hospital, Southeast University, Nanjing, China; University of Missouri-Columbia, United States of America

## Abstract

**Background:**

Microbubbles (MBs) can serve as an ultrasound contrast agent, and has the potential for magnetic resonance imaging (MRI). Due to the relatively low effect of MBs on MRI, it is necessary to develop new MBs that are more suitable for MRI. In this study, we evaluate the properties of SonoVue® and custom-made Fe_3_O_4_-nanoparticle-embedded microbubbles (Fe_3_O_4_-MBs) in terms of contrast agents for ultrsonography (US) and MRI.

**Methodology/Principal Findings:**

A total of 20 HepG2 subcutaneous-tumor-bearing nude mice were randomly assigned to 2 groups (i.e., n = 10 mice each group), one for US test and the other for MRI test. Within each group, two tests were performed for each mouse. The contrast agent for the first test is SonoVue®, and the second is Fe_3_O_4_-MBs. US was performed using a Technos^MPX^ US system (Esaote, Italy) with a contrast-tuned imaging (CnTI™) mode. MRI was performed using a 7.0T Micro-MRI (PharmaScan, Bruker Biospin GmbH, Germany) with an EPI-T_2_* sequence. The data of signal-to-noise ratio (SNR) from the region-of-interest of each US and MR image was calculated by ImageJ (National Institute of Health, USA). In group 1, enhancement of SonoVue® was significantly higher than Fe_3_O_4_-MBs on US (P<0.001). In group 2, negative enhancement of Fe_3_O_4_-MBs was significantly higher than SonoVue® on MRI (P<0.001). The time to peak showed no significant differences between US and MRI, both of which used the same MBs (P>0.05). The SNR analysis of the enhancement process reveals a strong negative correlation in both cases (i.e., SonoVue® r = −0.733, Fe_3_O_4_-MBs r = −0.903, with P<0.05).

**Conclusions:**

It might be important to change the Fe_3_O_4_-MBs' shell structure and/or the imagining strategy of US to improve the imaging quality of Fe_3_O_4_-MBs on US. As an intriguing prospect that can be detected by US and MRI, MBs are worthy of further study.

## Introduction

Angiogenesis is a determinant of tumor growth, invasion, and metastasis [1]. To detect new tumor microvessels, modern medical imaging modalities are widely used. As non-invasive imaging tools, ultrasonography (US) and magnetic resonance imaging (MRI) are becoming very popular.

With few exceptions, ultrasound contrast agents (UCAs) are microbubbles (MBs), which are often 1–7 µm in diameter and are used primarily as blood-pool markers [2]. Since the first description of enhanced reflections of ultrasound in 1969 [3], UCAs have developed rapidly and the existing MBs usually have an inert gas core (sulfur hexafluoride or perfluorocarbon gases) and a stable shell (denatured albumin, surfactants, or phospholipids). Typical MRI contrast agents include gadolinium chelate, manganese chelate, and iron compounds. However, in 1991, Moseley indicated that gas-filled MBs could also be used as a unique MR contrast agent [4]. Recent theoretical and phantom studies had further demonstrated this [5]–[9]. The principle behind their use in MRI was the gas-liquid interface or the pressure-induced microbubble size changing, which induced large local magnetic susceptibility differences.

The current research about MBs and MRI mainly falls into two categories: (1) using MBs as an MR contract agent based on the magnetic susceptibility of MBs [10]–[11], and (2) using MBs as a medium in MRI based on the biological effects produced by MBs' cavitation and sonoporation characteristics [12]–[14]. The first category of research has received little attention in the literature, mainly due to the relatively low effect of MBs on MRI. On the other hand, some researchers found that the potential application of MBs as a unique intravascular susceptibility contrast agent for MRI has not been fully studied. Along this line, references [4], [10] have focused on the feasibility study *in vivo* of MRI with existing MBs, and references [11], [15]–[19] mainly focused on developing new MBs that are more suitable for MRI.

In the present study, we are interested in determining the ability of microbubbles as contrast agents for ultrasonography and magnetic resonance imaging.

## Results

### *In vitro* US experiments

**Figure 1:**
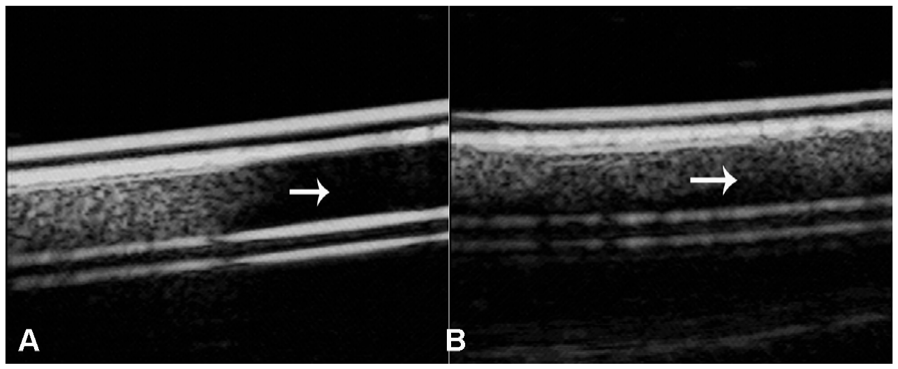
*In vitro* US experiments. Images of SonoVue® and Fe_3_O_4_-MBs under the mode of CnTI™ and Flash: (A) In the first half of the tube, in CnTI® mode, SonoVue® showed high echo; in the second half, in Flash mode, the SonoVue® microbubbles broke and the enhanced signal generated by the microbubbles changed to anecho (white arrow). (B) Under the same imaging strategy change, the signal of Fe_3_O_4_-MBs tube had little change (white arrow).

The signal strength was 92.08±7.45, 56.53±4.86 for SonoVue® and Fe_3_O_4_-MBs, respectively. There was a significant difference (P<0.001). When the imaging strategy changed from contrast-tuned imaging (CnTI™) to the Flash mode, the SonoVue® microbubbles broke and the enhanced signal generated by the microbubbles changed to anecho (the signal strength was 8.62±3.45, P<0.001, Fig. 1A). However, under the same imaging strategy change, the signal of Fe_3_O_4_-MBs tube had little change as few microbubbles broke in this case (the signal strength was 50.54±6.37, P>0.05, Fig. 1B).

### *In vitro* MRI experiments

**Figure 2:**
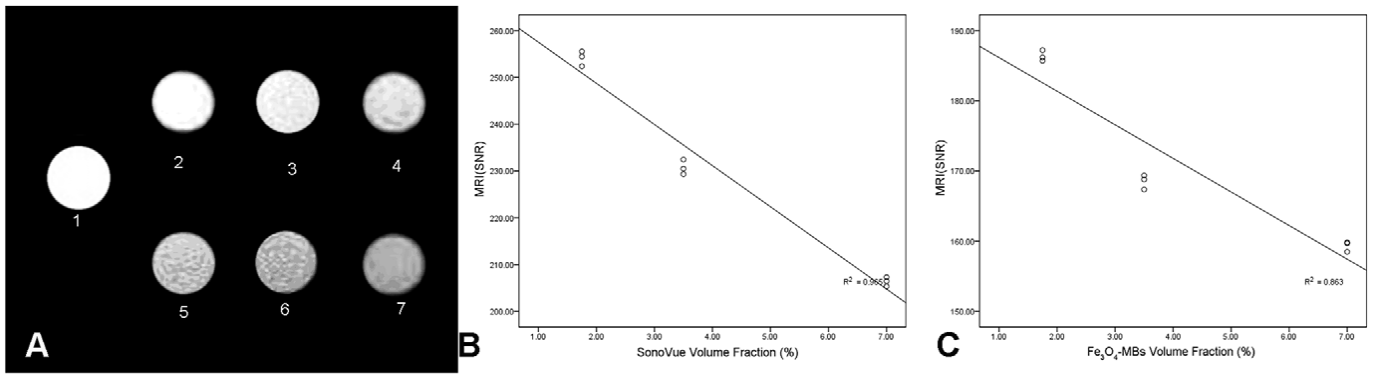
*In vitro* MRI experiments. The *in vitro* MR images of different volume fraction of SonoVue® and Fe_3_O_4_-MBs: (A). 1: sodium chloride solution (0.9% w/v), 2–4: SonoVue® with different volume fraction (1.75%, 3.5%, 7.0%). 5–7: Fe_3_O_4_-MBs with different volume fraction (1.75%, 3.5%, 7.0%). (B) an approximately linear relationship was observed in SonoVue® (r = −0.982, P<0.05) between SNR and microbubble volume fraction. (C) an approximately linear relationship was observed in Fe_3_O_4_-MBs (r = −0.929, P<0.05) between SNR and microbubble volume fraction.

Figure 2A includes MR images of different concentration in SonoVue® and Fe_3_O_4_-MBs suspension phantoms. The signal strength from Fe_3_O_4_-MBs was lower than SonoVue®. Figure 2B shows the dependency of the SNR on different SonoVue® volume fractions. Figure 2C shows the dependency of the SNR on different Fe_3_O_4_ -MBs volume fractions. An approximately linear relationship was observed independently (r = −0.982 for SonoVue®, r = −0.929 for Fe_3_O_4_-MBs, with P<0.05).

### *In vivo* US imaging

Generally, about 21 days after subcutaneous injection of tumor cells, the tumor maximum diameter was close to 0.77±0.08 cm. MB contrast enhancement was observed in all 10 mice by US and all 10 by MRI.

**Figure 3:**
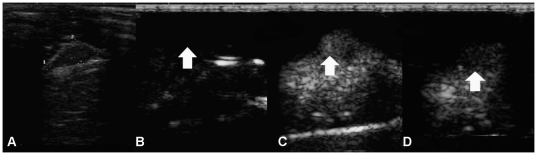
Representative US findings with SonoVue® and Fe_3_O_4_-MBs. Images from the same mouse: (A) gray-scale image of the tumor with a size 0.79 mm×0.34 mm, (B) under CnTI™ mode just before microbubble injection, signals from tumor was suppressed (white arrow), (C) maximum contrast enhancement US tumor image after SonoVue® injection (white arrow), and (D) maximum contrast enhancement US tumor image after Fe_3_O_4_-MBs injection (white arrow).

Figure 3 illustrates the images typically observed by US with SonoVue® and Fe_3_O_4_-MBs. Figure 3A shows the gray-scale image of the tumor. Under CnTI™ mode just before the MBs injection, signals from stationary tissues were suppressed and only high amplitude signals were visualized (Fig. 3B). After the MB suspension injection, the signal of the tumor enhanced. Figures 3C and 3D demonstrate the maximum contrast after injection SonoVue® and Fe_3_O_4_-MBs, respectively.

**Figure 4:**
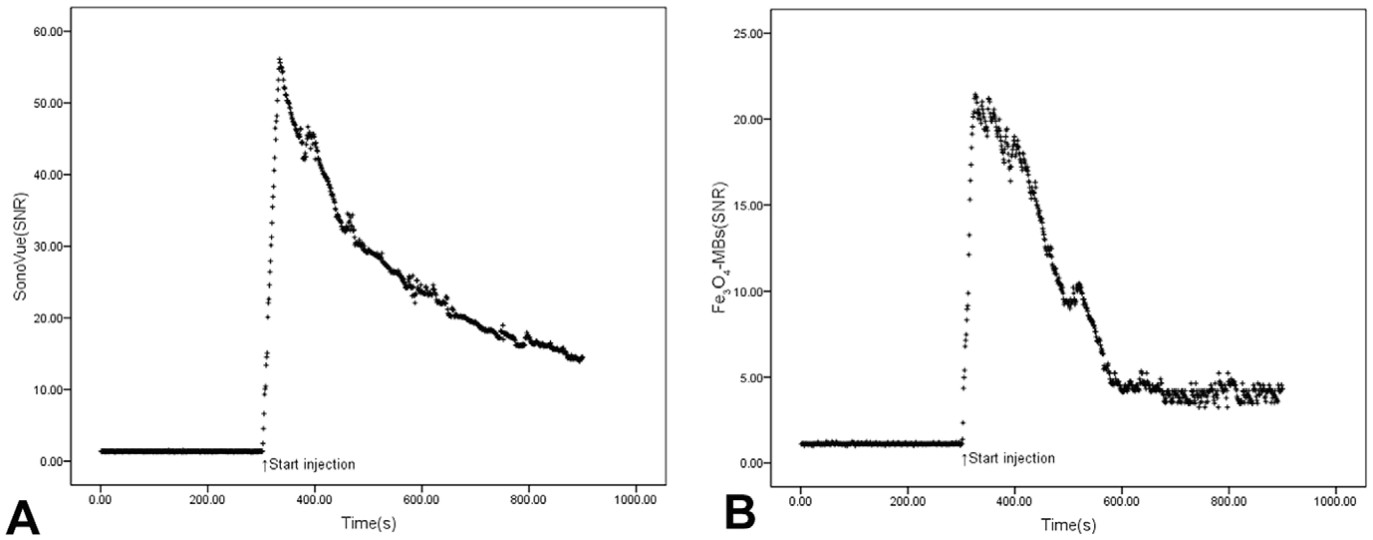
SNR time courses of the same mouse tumor in US by using SonoVue® and Fe_3_O_4_-MBs. Figure A shows the SNR time course in ROI during SonoVue® injection and Figure B shows the SNR time course when using Fe_3_O_4_-MBs injection.

Moreover, Figure 4 illustrates the time-course signal changes induced by SonoVue® (Figure 4A) and Fe_3_O_4_-MBs (Figure 4B) injection from the same region-of-interest (ROI) and the same tumor. The average value of enhanced signal observed was 26.14±10.95 and 8.52±5.83 for SonoVue® and Fe_3_O_4_-MBs during the entire imaging process, respectively, and there was a significant difference (P<0.001).

### *In vivo* MR imaging

**Figure 5:**
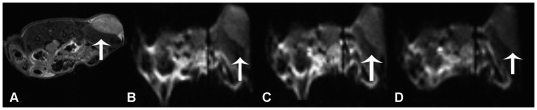
Representative MRI findings with SonoVue® and Fe_3_O_4_-MBs. Images from the same mouse during microbubble injection: (A) anatomical image showing the superficial tumor (white arrow), (B) pre-injection EPI-T_2_* MRI tumor image (white arrow), (C) maximum contrast enhancement of the tumor after SonoVue® injection (white arrow), and (D) maximum contrast enhancement of the tumor after Fe_3_O_4_-MBs injection (white arrow).

Typical time courses of EPI-T_2_* images show the similar change trends as US after MBs administration. Figure 5 illustrates the mouse tumor images typically observed during SonoVue® and Fe_3_O_4_-MBs injection. Figure 5A shows the anatomy of superficial tumor. Figure 5B illustrates pre-injection EPI-T_2_* images. The post-injection images of SonoVue® and Fe_3_O_4_-MBs correspond to the lowest SNR point in Figures 5C and 5D.

**Figure 6:**
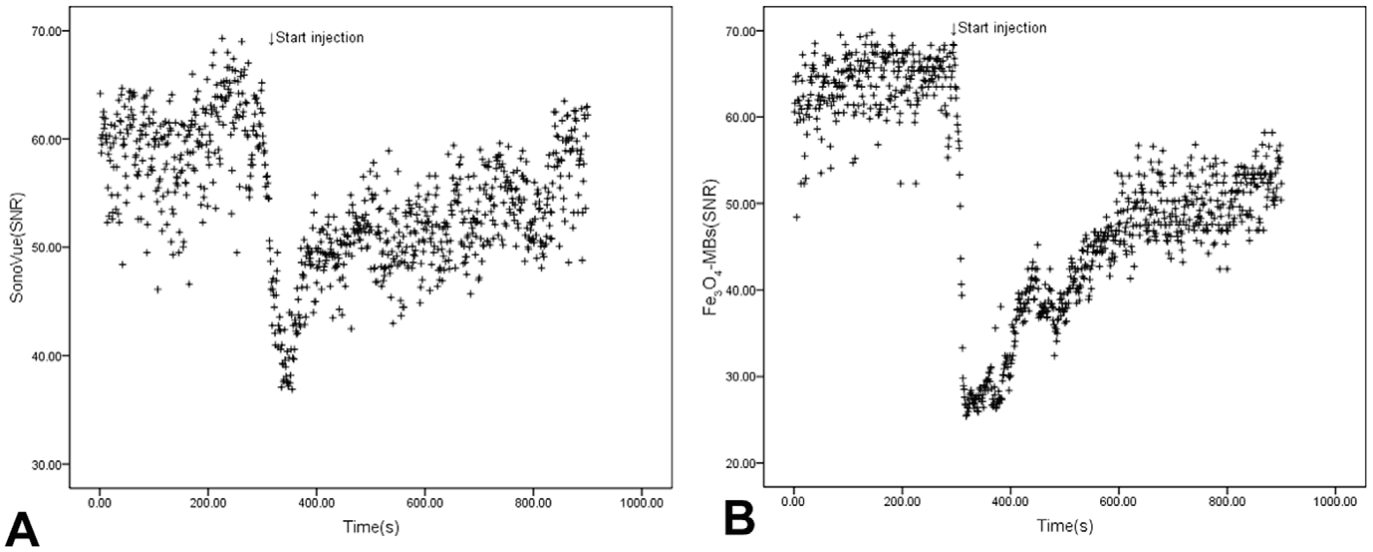
SNR time courses of MRI from SonoVue® and Fe_3_O_4_-MBs. Figure A shows the SNR time course in ROI during SonoVue® injection and Figure B shows the SNR time course when using Fe_3_O_4_-MBs.

Moreover, Figure 6 illustrates the SNR time-course signal changes induced by SonoVue® (Figure 6A) and Fe_3_O_4_-MBs (Figure 6B) injection from the same ROI and the same tumor. The average signal strength was 51.57±5.01 and 43.80±8.38 for SonoVue® and Fe_3_O_4_-MBs respectively, and there was a significant difference (P<0.001).

### Correlation between the US and MRI

Time-to-peak values were 33.60±1.58 s and 34.20±1.55 s in SonoVue® for US and MRI, respectively, and there was no significant difference (P = 0.402). For Fe_3_O_4_-MBs, the corresponding values are 26.50±1.27 s and 23.80±1.69 s for US and MRI, respectively, and there was no significant difference either (P = 0.404).

**Figure 7:**
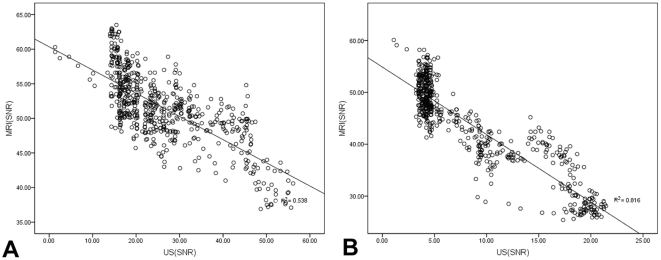
Correlation between the US and MRI. Figure A shows the signal strength correlation between US and MRI in 600 seconds of enhancement when using SonoVue® (r = −0.733, P<0.05). Figure B shows the signal strength correlation between US and MRI in 600 seconds of enhancement when using Fe_3_O_4_-MBs (r = −0.903, P<0.05).

Figure 7A denotes the negative correlation of the tumor signal strength change between US and MRI when both using SonoVue®. The corresponding correlation is r = −0.733 with p<0.05. Figure 7B shows a similar negative correlation when we use Fe_3_O_4_-MBs in US and MRI instead. The corresponding correlation in this case is r = −0.903 with p<0.05. The above SNR analyses revealed a strong and significant relationship between the two modalities when using the same microbubble contrast agent.

## Discussion

Despite the enhancement in different ways, the two typical SNR time courses showed the similar trend. The tumors were enhanced rapidly first and washed out gradually. The time of dynamic enhancement was only a few minutes after injection because of the limited lifetime of MBs *in vivo*.

In group 1, Fe_3_O_4_-MBs had a lower effect on US than SonoVue®. The reasons are mainly (1) the Fe_3_O_4_-MBs is a relatively new material and has a hard shell, their acoustic properties (e.g., acoustic impedance) are somewhat different from phospholipid-stabilized MBs under the same acoustic energy, so the intensity of second harmonic is relatively weak. (2) Most of the medical equipment today has been designed and tested based on the properties of SonoVue®. Without changing the imaging strategies, it is difficult to achieve good imaging properties with the new Fe_3_O_4_-shelled microbubbles. We have conducted many tests to improve the imaging quality on the existing medical devices. However, methods such as using higher frequencies or Doppler based destructive imaging cannot achieve the desirable effects yet. We are working with several Chinese equipment manufactures on improving the imaging qualities, hopefully obtaining some positive results soon.

In group 2, however, SonoVue® had a lower effect on MRI than Fe_3_O_4_-MBs, and the effect of SonoVue® had reached its limit according to the approaches described for improving microbubbles for MRI, which included increasing the radius and volume fraction of MBs, using inert gas core, et al [8], [10]. First, as the size SonoVue® ranges from 0.1 µm to 1.1 µm (with a mean diameter is 2.5 micrometer according to manual), thus the diameter should not be increased significantly or they will not pass through the pulmonary circulation. Second, the microbubble concentration used in our study was 0.1 mL of the ∼3.5% volume fraction, which was far more than the common maximum clinical dosage (e.g., 0.08 mL/kg of the 0.8% volume fraction according to the product description) [10]. Third, the gas core is already sulfur hexafluoride. Since an inert gas has a high molecular weight and low solubility, it can cause the largest effect on transverse relaxation.

Comparing with SonoVue®, the most important change in the custom-made MBs was that some Fe_3_O_4_ nanoparticles had been embedded into a double-polymer shell. Fe_3_O_4_ are T_2_ agents as MBs, and they can enhance the magnetic sensitivity for the MBs [11], [18]. As the thick shell [20], the susceptibility of US is reduced. Changing the characteristics of the shell and/or the imagining strategy on US may be the most important factor in improving the effectiveness for US. After all, a combination of multiple modalities can offer synergistic advantages over any modality alone [21].

In a closely related paper [22], the authors reported an early contrast study of tumor perfusion using US and MRI. They used MBs as the contrast agent for US and gadopentetate dimeglumine as the contract agent for MRI. And found that using MBs in US can achieve the same effect as using gadopentetate dimeglumine in MR imaging. As an intriguing prospect that can be detected by US and MRI, MBs are worthy of further study.

## Materials and Methods

### *In vitro* US experiments

A microbubble phantom study was performed (L.L., Q.W., H.B.L.) to measure SNR of SonoVue® and Fe_3_O_4_-MBs suspension using a Technos^MPX^ US system (Esaote, Italy) with a linear-array transducer (LA532E, 7.5 MHz for fundamental gray-scale imaging and 2.5 MHz for CnTI™). Fe_3_O_4_-MBs was kindly provided by Dr. Fang Yang [18], which had double polymer shells with 86.47 µg/mL Fe_3_O_4_ nanoparticles in it and N_2_ gas core and the mean diameter was 3.98 µm. The samples were well mixed and placed in silica gel tubes of 1.0 cm in diameter. They were diluted to 3.5% volume fractions with sodium chloride solution (0.9% w/v) and put in a de-gassed water tank. Two experiments were conducted, one under CnTI™ imaging and the other under Flash imaging. The CnTI™ parameters were as follows: the gain was 105, the depth was 31 mm, and the mechanical index (MI) was 0.089, while in the Flash mode the MI was 0.5.

### *In vitro* MRI experiments

A microbubble phantom study was performed (L.L.,S.W.) to measure SNR of Fe_3_O_4_-MBs and SonoVue® suspension using a 7.0T Micro-MRI (PhamaScan, Bruker, Germany). The samples were mixed and placed in Eppendorf tubes of 1.0 cm in diameter. They were diluted to 7.0%, 3.5%, and 1.75% volume fractions with sodium chloride solution (0.9% w/v) respectively. Every phantom was scanned three times. The imaging parameters for T_2_-weighted fast spin echo were set as repetition time (TR) = 3000 ms, echo time (TE) = 60 ms, number of excitations (NEX) = 1.

### Microbubble preparation for *in vivo* experiments

The first contrast agent used was SonoVue®. The second contrast agent used was Fe_3_O_4_-MBs. For each group, the dosages are all 0.1 mL of microbubble suspension with a volume fraction of about 3.5%. Before each injection, re-suspension must be performed.

### Cell culture and animal model

All animal procedures were performed in accordance with the approval and guidelines of the Institutional Animal Care and Use Committee (IACUC) of the Medical School of Southeast University (approval ID: SYXK-2007.2121).

Cells from the human hepatocellular liver carcinoma cell line HepG2 (Keygen Biotech. Co., Ltd, Nanjing, China) were grown in RPMI 1640 with 10% fetal bovine serum (FBS) in a 5% CO_2_ humidified atmosphere at 37°C. Tumors were established in 20 healthy BALB/c-nu mice (5–6 weeks old, 20–25 g in weight) in random order by subcutaneous injection of a suspension of 2×10^7^ HepG2 cells in 0.2 mL of phosphate-buffered saline (PBS) in the right leg. Tumors were allowed to grow until the greatest diameter of the tumors was close to 0.8 cm.

### Animal preparation

A total of 20 HepG2 subcutaneous-tumor-bearing nude mice were randomly assigned to 2 groups (i.e., n = 10 mice each group), one for the US test, and the other for the MRI test. Within each group, two tests were performed for each mouse. The contrast agent for the first test is SonoVue®, and the second is Fe_3_O_4_-MBs. For US, all mice were kept anesthetized with intraperitoneal injection of 10% chloral hydrate (3 mL/kg). For MRI, mice were anesthetized with 1.0% isoflurane via a nose core with respiratory monitoring. A 30-G needle was inserted into a tail vein by intravenous injection of the bubbles with a 40-cm long PE-10 tube. The dose of MBs was 0.1 mL and 0.2 mL of saline flush was administered immediately after MBs injected.

### *In vivo* US experiments

Before each examination, each mouse was fixed in a left lateral position on a warm pad to maintain the body temperature. As the tumor was just under the skin, about a 2-mm deep US gel was placed between the transducer surface and the skin to ensure the tumor was imaged clearly by US.

US imaging was performed (L.L., Q.W., H.B.L.) using a Technos^MPX^ US system under CnTI™ mode. The CnTI™ parameters were maintained during all examinations: the gain was 105, the depth was 31 mm, the MI was 0.089 and the scan time was 1 s. When the optical imaging plane was obtained (the tumor's largest transverse cross section), the transducer was maintained with a mechanical fixer. MB suspension was injected about 5 min after the CnTI™ was triggered and images were recorded digitally on a hard disk for 15 min for off-line analysis. The data of SNR from the ROI of each US image was calculated by ImageJ (a software by National Institute of Health, USA). The time-course of the corresponding parameter SNR was measured.

For each mouse, SonoVue® check was done first. After the sufficient clearance of the MBs, Fe_3_O_4_-MBs suspension was injected.

### *In vivo* MRI experiments

MRI was performed (L.L., S.W.) using a 7.0T Micro-MRI with a 38-mm volume coil. Each mouse was placed in the prone position and dynamic susceptibility imaging was performed using an EPI-T_2_* sequence with the respiratory gating control. To obtain better images and shorten the scan time as much as possible, parameters were adjusted as follows: TR = 1000 ms; TE = 30 ms; field of view (FOV) = 4×4 cm; FA = 90°; NEX = 1; scan time = 1 s; slice thickness = 1 mm. Anatomical images were acquired under another protocol using the following parameters: TR = 2500 ms; TE = 33 ms; FOV = 4×4 cm; FA = 180°; NEX = 1; scan time = 1 min20 s; slice thickness = 1 mm. MB suspension was injected about 5 min after the start of the dynamic imaging and the total scanning time was 15 min. All images were recorded digitally on a hard disk for off-line analysis. ROI analyses were conducted as described for US and the SNR time course was also measured.

For each mouse, SonoVue® check was done first then was the Fe_3_O_4_-MBs.

### Statistical analyses

Statistical evaluation was performed using SPSS software (ver.13.0; SPSS Inc., Chicago, IL, USA). The numerical results were expressed as means±SD. Results were analyzed statistically using the paired-samples t test (for comparing the average signal strengths of using different MBs in US or MRI) and independent-samples t test (for comparing the time-to-peak average signal strengths of using the same MBs in US and MRI) for effectiveness of different MBs and modalities. Pearson correlation coefficient of SNR is for US and MRI.
